# Genetic structure in the paternal lineages of South East Spain revealed by the analysis of 17 Y-STRs

**DOI:** 10.1038/s41598-019-41580-9

**Published:** 2019-03-26

**Authors:** María Saiz, Maria Jesus Alvarez-Cubero, José Antonio Lorente, Juan Carlos Alvarez, Luis Javier Martinez-Gonzalez

**Affiliations:** 1 University of Granada, Laboratory of Genetic Identification, Department of Legal Medicine, Toxicology and Physical Anthropology, Faculty of Medicine, PTS. Avd. Investigación 11, 18016 Granada, Spain; 2 University of Granada, Department of Biochemistry and Molecular Biology III and Inmunology, Faculty of Medicine, PTS. Avd. Investigación 11, 18016 Granada, Spain; 3 GENYO, Centre for Genomics and Oncological Research: Pfizer/University of Granada/Andalusian Regional Government, PTS. Avenida de la Ilustración, 114, 18016 Granada, Spain

## Abstract

The genetic data of 17 Y chromosome short tandem repeats in 146 unrelated donor residents in the provinces of Granada, Málaga, and Almería (GMA) were analyzed to determine the genetic legacy of the male inhabitants of the former Kingdom of Granada. A total of 139 unique haplotypes were identified. Observed allele frequencies and haplogroup frequencies were also analyzed. By AMOVA and STRUCTURE analysis, the populations of the 3 provinces could be treated genetically as a single population. The most frequent haplogroup was R1b1b2 (58.22%). By network analysis of all individuals, we observed a distribution according to haplogroup assignment. To improve the characterization of GMA population, it was compared with those of North Africa, the Iberian Peninsula, and southern Europe. In our analysis of allele frequencies and genetic distances, the GMA population lay within the Spanish population group. Further, in the STRUCTURE analysis, there was no African component in the GMA population, confirming that, based on our genetic markers, the GMA population does not reflect any male genetic influence of the North African people. The presence of African haplogroups in the GMA population is irrelevant when their frequency is compared with those in other European populations.

## Introduction

For the nearly 800 years of the Arab invasion of the Iberian Peninsula, North African groups spread throughout all the territory except for the Basque Country, Galicia, Cantabria, Asturias, and most of the Pyrenees; but, their influence was greater in the south of the Peninsula^1^. The former Kingdom of Granada comprised the present-day provinces of Granada, Málaga, and Almería, in addition to parts of Cádiz, Jaén, Córdoba, and Sevilla, with Granada as the capital. During the 14th and 15th centuries, it was one of the most prosperous cities in Europe, with almost 165,000 inhabitants^2^.

The first evidence of African invaders originated from 711, when Syrian Berbers entered the Iberian Peninsula and conquered the Granada region, known as *Iliberir*. In 1013, the Zirid dynasty gained control of the region and established *Ilbira* in 1025. To prevent future invasions, the kingdom expanded and occupied the entire territory of the Kingdom of Granada^3,4^. With the expansion of the Kingdom in 1090, a large part of the Iberian Peninsula came under control of the Zirid dynasty, known as *Al-Ándalus*. After losing the battle of Navas de Tolosa, in 1212, *Al-Ándalus* became subdued and was reduced to the Nasrid Kingdom of Granada. The Nasrid dynasty was the longest surviving Muslim dynasty in the Iberian Peninsula. Ultimately, with the confiscation of the city of Granada by *Los Reyes Católicos*, *Fernando II*, and *Isabel I* in 1492, the Kingdom of Granada came to an end^3,4^.

Although the Muslims signed capitulation agreements to follow the religion of the kingdom, they were forced to convert to Christianity or emigrate. Once the Morisco properties were expropriated and expelled, it became necessary to repopulate the region by sending new inhabitants from various regions of the Peninsula. The repopulation began in 1571 and lasted until 1595, by which time a total of 12,546 families repopulated 270 areas^3^. On the December 9, 1609, *Felipe III* signed the expulsion warrant of all Moriscos from Spain^3,4^. In 1833, after 314 years of existence and with the separation of the provinces of Almería and Málaga, the former Kingdom of Granada ended^3,4^.

Thus, during the establishment of the Kingdom of Granada and throughout its existence, people from diverse religions and regions coexisted. The coexistence of Visigoths, Syrians, Saharans, Moroccans, Jews, and Christians is reflected in the architecture, culture, and folklore of the present-day cities of Granada, Málaga, and Almería.

The study of mtDNA and Y chromosomes has identified geographic regions with a genetic influence of North Africans of 8% to 10%^5–7^. Crossbreeding studies that are based on the characterization of ALU sequences have found traces of sub-Saharan genes in north Mediterranean populations, suggesting continuous contact between both coasts^8^. That genetic traits and certain specific haplotypes have been detected along the northern coast of the Mediterranean Sea confirms the hypothesis that gene flow in this region was linked to the first trans-Mediterranean sailings and remained homogeneous while the slave trade lasted, until the late 17th century, rather than reflecting Islamic expansion (S. VII to S. XV)^8^. Y chromosome studies have described the genetic structure in the Iberian Peninsula, calculating the various contributions of Muslims and Jews to the current population of the Peninsula^6^, focusing on the E3b2 haplogroup, which is common in northern Africa and present in 5.6% of the Peninsula^9^. The distribution of the Y chromosome haplogroup E-M81 in the Iberian Peninsula suggests genetic flow of North Africa during this period^6^. High levels of patrilineal descent from North Africa and Sephardic Jews in the current population of the Iberian Peninsula have been detected^6^ and contributed to the higher genetic diversity in southwestern Europe^10^.

However, contrary to what might be expected based on historical data that favor a gradient of North African genetic influence from south to north, most such influence has been found in Galicia and northern Castilla (>20%)^6^. The main gradient of the frequencies of North African genes descend from west to east^11^. Furthermore, recent studies based on autosomal SNPs^11^ and Y-chromosome lineages^12^ reveal that Andalusian population does not specially cluster with North African populations more than other Iberian populations^13^. After the Reconquest, the Moors were distributed homogeneously throughout the Peninsula, but their final expulsion in 1609 was absolute in certain regions of Spain, Valencia, and western Andalusia, whereas in Galicia and Extremadura, the population dispersed and integrated into society^6^.

## Results

### Y-STR allele frequencies

Allele frequencies and forensic summary statistics for the 16 loci are summarized in Table 1. The most informative markers were DYS385 and DYS458, and the least informative marker was DYS393. The combined discriminatory power was 1–6.17168·10^−8^. A total of 139 unique haplotypes were detected, 133 of which were described once, 5 that were described twice, and 1 that was described 3 times. The Y chromosome haplotype diversity was 0.9974, and the discriminatory capacity was 95.21% [(N haplotypes/n)·100].

**Table 1:** Allele frequencies and forensic summary statistics of the Yfiler STR loci found in the GMA population sample.

Allele	DYS456	DYS389I	DYS390	DYS389II	DYS458	DYS19	DYS393	DYS391	DYS439	DYS635	DYS392	GATA_H4	DYS437	DYS438	DYS448	Allele	DYS385 A/B
**8**								0.007						0.007		**10**.**14**	0.014
**9**								0.068				0.014		0.110		**11**.**11**	0.034
**10**								0.548	0.082		0.007	0.034		0.274		**11**.**13**	0.041
**10**,**11**									0.014							**11**.**14**	0.336
**11**								0.363	0.301		0.336	0.411		0.055		**11**.**15**	0.062
**12**	0.014	0.130					0.171	0.014	0.466		0.062	0.473		0.534		**11**.**16**	0.007
**13**	0.027	0.589				0.144	0.719		0.137		0.541	0.062		0.021		**11**.**18**	0.007
**14**	0.041	0.274			0.048	0.644	0.110				0.041	0.007	0.459			**12**.**12**	0.034
**15**	0.486	0.007			0.103	0.158					0.014		0.479			**12**.**13**	0.007
**16**	0.349				0.144	0.034							0.062			**12**.**14**	0.062
**16**.**2**					0.007											**12**.**15**	0.014
**17**	0.055				0.370	0.021										**12**.**16**	0.007
**17**.**2**					0.034											**13**.**13**	0.007
**18**	0.027				0.178										0.166	**13**.**14**	0.055
**18**.**2**															0.007	**13**.**15**	0.021
**19**					0.068										0.503	**13**.**16**	0.048
**20**					0.048					0.075					0.207	**13**.**18**	0.014
**21**			0.021							0.205					0.090	**13**.**19**	0.021
**22**			0.068							0.116						**14**.**14**	0.041
**23**			0.301							0.486					0.028	**14**.**15**	0.014
**24**			0.541							0.110						**14**.**16**	0.034
**25**			0.068							0.007						**14**.**18**	0.007
**27**				0.021												**14**.**19**	0.007
**28**				0.096												**15**.**15**	0.014
**29**				0.432												**15**.**16**	0.007
**30**				0.349												**15**.**17**	0.014
**31**				0.082												**15**.**18**	0.007
**32**				0.021												**15**.**19**	0.007
																**16**.**17**	0.014
																**16**.**18**	0.007
																**18**.**18**	0.007
**PD**	0.635	0.561	0.607	0.676	0.790	0.538	0.441	0.563	0.667	0.690	0.589	0.603	0.556	0.624	0.667		0.864
**PIC**	0.573	0.495	0.546	0.619	0.766	0.498	0.398	0.480	0.611	0.652	0.518	0.523	0.456	0.570	0.624		0.859
**MP**	0.365	0.439	0.393	0.325	0.210	0.462	0.559	0.437	0.333	0.310	0.411	0.397	0.444	0.376	0.333		0.136

### Population substructure

By AMOVA, there was no significant genetic substructure in the GMA population (p > 0.05). All variations were within populations (99.93%) rather than between populations (0.07%) (Table 2).

**Table 2:** AMOVA design and results from 146 individuals (70 from Granada, 42 from Málaga and 34 from Almería).

Structure design	Source of variation	d.f.	Sum of squares	Variance components	Percentage of variation
3 Groups:	Among populations	2	1.032	0.00036 Va	0.07
Group 1 = Granada Group 2 = Málaga Group 3 = Almería	Within populations	143	71.413	0.49939 Vb	99.93
	Total	145	72.445	0.49975	Fixation index FST: 0.00071

### Y chromosome haplogroups

Based on the 17 Y chromosome STR markers, most men in the GMA population carried the R1b1b2 haplogroup (58.22%). The second most frequent haplogroup was E1b1b1; many subhaplogroups were detected (11.64%), of which E1b1b1b was the most frequent (4.79%). J2a4 had a proportion of 6.16%. Other subhaplogroups from J included J1c3 (4.11%) and J1-M267, J2b1, and J2bf2 (0.68%). The G2a-P15 haplogroup was observed in 5.48% of samples (Table 3).

**Table 3:** Y chromosome haplogroup frequencies in the GMA population.

_IJ M429	E1b1a1g U175	E1b1b1 M35	E1b1b1a1b V13	E1b1b1a1cV22		E1b1b1a1d V65	E1b1b1bM81	E1b1b1cM123
0.0068	0.0068	0.0068	0.0205	0.0137		0.0137	0.0479	0.0137
**F-M89**	**G2a-P15**	**H1a-M82**	**I2a-P37**.**2**	**I2a2a-M223**		**I2a2-S154**	J1*-M267*	**J1c3-P58**
0.0068	0.0548	0.0068	0.0205	0.0274		0.0068	0.0068	0.0411
**J2a4 L26**	**J2b1 M205**	**J2b2f-L283**	**P-M45**	**Q1a MEH2**	**Q1b M378**	**R1b1b2-M269**	**T1a1a1 P77**	**T1a2 L131**
0.0616	0.0068	0.0068	0.0068	0.0137	0.0068	0.5822	0.0068	0.0068

**Figure 1:**
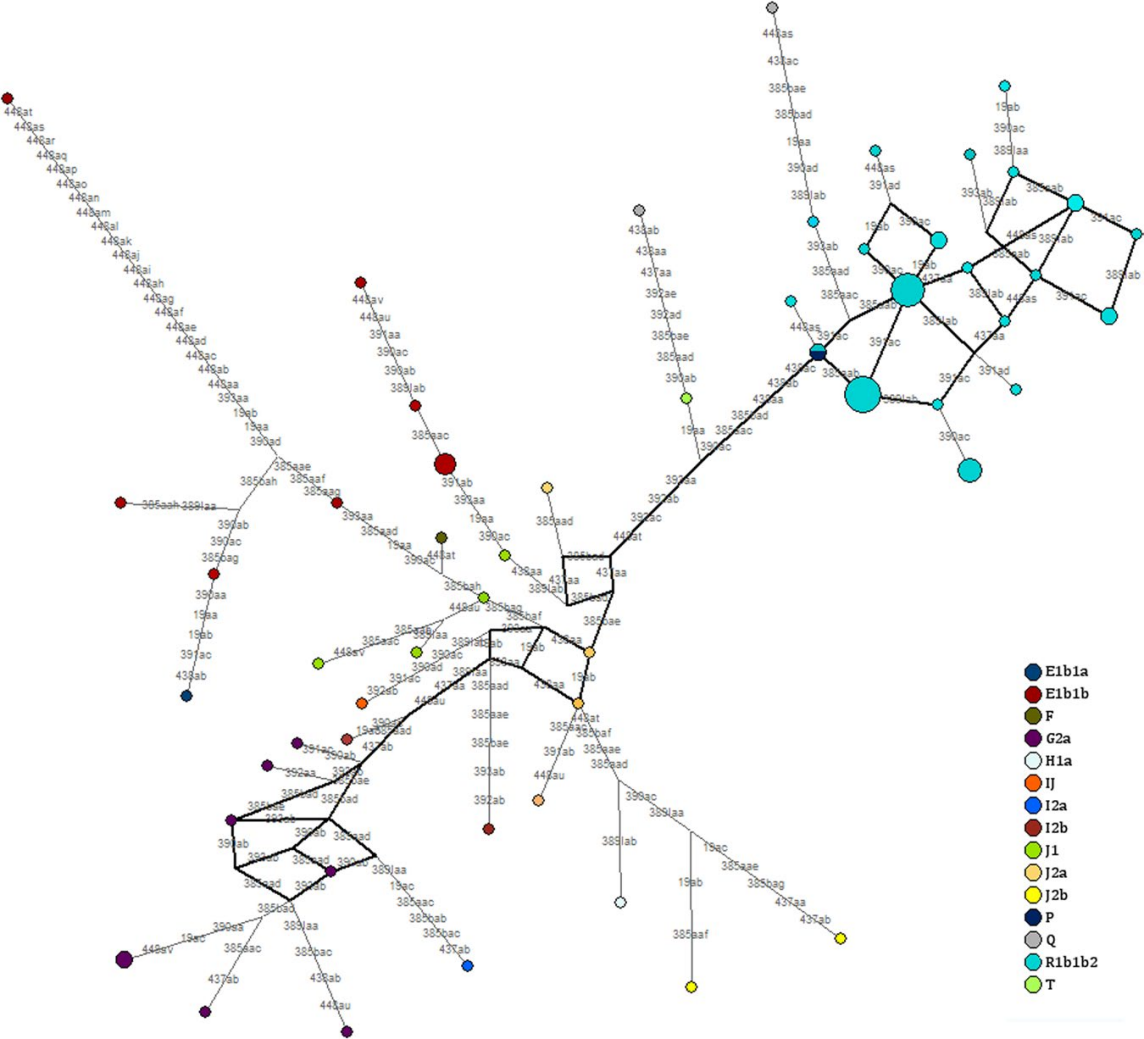
Network for Y chromosome haplogroups.

Network analyses were performed for all haplogroups (n = 79) (Fig. 1) and those in the R1b1b2 haplogroups (n = 85) (Supplementary Fig. 1), derived from the data on 10 markers with mutation rates under 0.0025 (DYS19, DYS389I, DYS390, DYS391, DYS392, DYS393, DYS385, DYS438, DYS437, and DYS448). Within both networks, no portioning of populations was observed by province, and individuals from the 3 provinces were distributed randomly throughout the network.

Individuals were then distributed by haplogroup (Fig. 1). The central node of the network contained the G2a, J2a, and R1b1b2 haplogroups. All individuals from the R1b1b2 haplogroup converged in 2 nodes (8 and 9 individuals), after which the remainder of the groups was created.

### Surname analysis

Twenty repeated surnames were identified among the 108 surnames of the 146 individuals (58 individuals), most of which were associated with the R1b1b2-M269 haplogroup (28/58). Haplogroups from lineages E (E1b1b1c-M123, E1b1b1a1b-V13, E1b1b1b1b-M81, E1b1b1*-M35, E1b1b1a1d-V65, E1b1a1g-U175, and E1b1b1a1c-V22), I (I2a-P37.2 and I2b1-M233), J (J2a4-L26, J2b2f-L283, and J1c3-P58), and G2a-P15 were also common between surnames. Table 4 shows the composition of Y chromosome haplogroups for the 4 most frequent surnames in the sample and for double and triple surnames. R1b1b2-M269 was the most common haplogroup in this surname set, although other common European and North African haplogroups were detected. In addition, 6 Spanish surnames of Arab origin were detected, all of which were singletons—5 were linked to Y chromosome R1b1b2-M269, whereas the remainder was associated with the J1c3-P58 lineage. Network analysis on these 6 individuals did not reveal any one of them to be a specific ancestor of the other subjects (data not shown).

**Table 4:** Y chromosome haplogroup frequencies in surnames with more than 1 occurrence in the Andalusian sample.

	Doubletons	Trios	Rodríguez	García	Sánchez	Fernández
n	20	18	4	5	5	6
E1b1b1*-M35(xM78.M81.M123)	0.05					
E1b1b1a1b-V13	0.05	0.056				
E1b1b1b-M81	0.1					0.167
E1b1a1g-U175			0.25			
E1b1b1a1c-V22			0.25			
E1b1b1c-M123	0.05	0.056				
E1b1b1a1d-V65		0.056				
I2a-P37.2	0.05	0.056			0.2	
I2b1-M223	0.05	0.111			0.2	
F-M89		0.056				
G2a-P15	0.05				0.2	
H1a-M82			0.25			
J2a4-L26	0.05	0.056		0.2		
J2b2f-L283						0.167
J1c3-P58						0.333
Q1a-MEH2	0.05					
R1b1b2-M269	0.45	0.556	0.25	0.8	0.4	0.333
T1a1b-P77	0.05					

Unrelated men who have the same surname are significantly more likely to share haplotypes^14,15^. However, in the GMA population, none of the individuals who shared haplotypes had the same surname. Further, except for Martín and Gomez, the remaining individuals who shared haplotypes had unique surnames. Six of the 108 different surnames in the GMA population were Arab in origin; only Silla was associated with the J1c3-P58 lineage, a characteristic of the haplogroup in the population of the Arabian Peninsula.

### Population cross-comparisons

**Figure 2:**
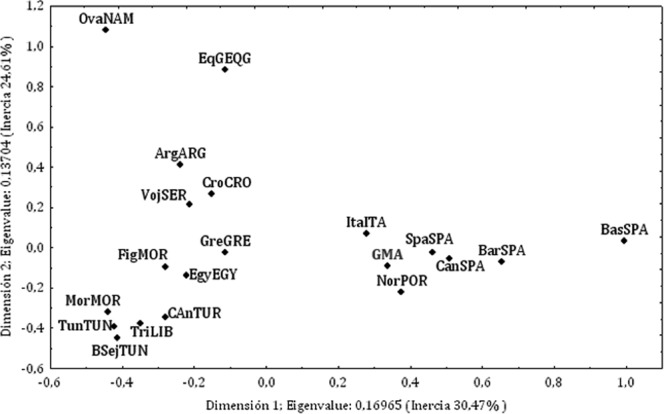
Correspondence analysis between GMA population and European and African populations (MorMOR, Morocco; FigMOR, Figuig Oasis from Morocco; ArgARG, Oran Area from Algeria; BSejTUN, Berbers from Sejenane from Tunisia; TunTUN, Tunisia; EgyEGY, Egypt; TriLIB, Tripoli Region from Libya; CAnTUR, Central Anatolia from Turkey; BasSPA, Basque Country from Spain; CanSPA, Cantabria from Spain, BarSPA; Barcelona from Spain, SpaSPA, Spain; NorPOR, North of Portugal; ItaITA, Italy; GreGRE, Greece; CroCRO, Croatia; VojSER, Vojvodina from Serbia; HolHOL, Holland; EqGEQG, Equatorial Guinea; OvaNAM, Ovambo from Namibia).

Y chromosome allele frequencies from the GMA population and published frequencies (Supplementary Table 1) were used to perform correspondence analysis (Fig. 2). The first 2 main components, together accounting for >50% of the total variance, suggested proximity of the GMA population to Spanish and European populations, clearly occupying the same genetic space.

Two main groups were observed in the analysis of Y chromosome allele frequencies (Fig. 2). One comprised the Iberian Peninsula and Mediterranean populations. Due to the frequencies of marker DYS438 (alleles 12 and 13) and marker DYS392 (allele13), the Basque Country population^16^ lay far from the rest of the Spanish populations^16,17^. The other group was composed of North African populations^18–23^; the Algerian population^20^ resided outside of this group.

**Figure 3:**
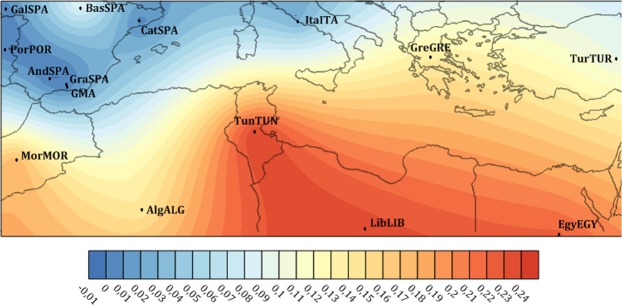
Map of Y STR RST genetic distances between the GMA population and Mediterranean and North African populations plotted with Surfer 13 using the Kriging method dark blue corresponds to lower values of genetic distances and dark red to higher values of genetic distances (MorMOR, Morocco; AlgALG, Oran Area from Algeria; TunTUN, Tunisia; EgyEGY, Egypt; LibLIB, Libya; TurTUR, Turkey; BasSPA, Basque Country from Spain; GalSPA, Galicia from Spain, CatSPA; Catalonia from Spain, AndSPA, Andalusia from Spain; GraSPA, Granada from Spain; PorPOR, Portugal; ItaITA, Italy; GreGRE, Greece).

The genetic distances confirm the results of the genetic frequency analysis (Fig. 3). When Rst values were visualized with Surfer, the populations with the closest affinity to the GMA population (populations within the blue gradient lines) were from other regions of the Iberian Peninsula^24,25^ and nearby Mediterranean populations^26–31^. The Libyan and Tunisian populations had the biggest differences compared with the GMA populations (darker red gradient). Studies in the Libyan population support the importance of migratory movements that lead to admixture between the original Berber inhabitants and neighboring and more distant populations, despite a solid Berber genetic background remaining^32^. Berber populations inhabited the Iberian Peninsula for almost 250 years^3^ but were completely expelled, and very few genetic substrata can be detected in the GMA population, as evidenced by the high genetic differences between the GMA population and those with a significant Berber genetic influence.

The Rst pairwise distances between the GMA and European and African populations indicated that prior to Bonferroni adjustments; all pairwise comparisons generated statistically significant differences.

## Discussion

The populations of the provinces of Granada, Malaga, and Almeria behaved as a single population with regard to their genetic structure. These results are consistent with the historical and cultural expectations, because these 3 provinces share the same origin and proximity over a large area. We are aware that 146 individuals is a small sample size for 3 provinces. Reduced sample sizes in population and evolutionary studies may underscore the allelic frequencies of certain alleles, underestimating the number of alleles that aredetected^33,34^. However, studies that have compared different samples sizes have revealed that, after increasing sample size, newly detected alleles are rare^34^. In this case, nearly all alleles represented in the allelic ladder are represented in the population and all alleles have frequencies that are similar to those in nearby populations^24^.

Based on the analysis of 17 Y chromosome STR markers, most men in the GMA population carried the R1b1b2 (haplogroup 58.22%), which is the most common European haplogroup, increasing in frequency east to west. This gradient indicates that this haplogroup spread throughout Europe from a single source in the Near East during the Neolithic period^35^. The second most frequent haplogroup was E1b1b1, but many subhaplogroups were seen (11.64%), of which E1b1b1b was the most frequent (4.79%). The highest frequencies of this haplogroup were detected in the north of Africa, at >80% in the Moroccan Berbers^36^. However, this haplogroup is rare in Europe, except in the Iberian Peninsula (5% of the individuals)^6,36^. Studies based on whole Y-chromosome sequences attribute these frequencies to genetic drift acting on this low-frequency variant^37^. J2a4 had a proportion of 6.16%. The other subhaplogroups from J that were observed were J1c3 (4.11%), typically seen in Arabian Peninsula populations (40% to 75% male lineages)^38^ and J1-M267, J2b1, J2bf2 (0.68%)—both lineages (J1 and J2) are detected primarily in southeast Europe. The G2a-P15 haplogroup was observed in 5.48% of samples (Table 2).

**Figure 4:**
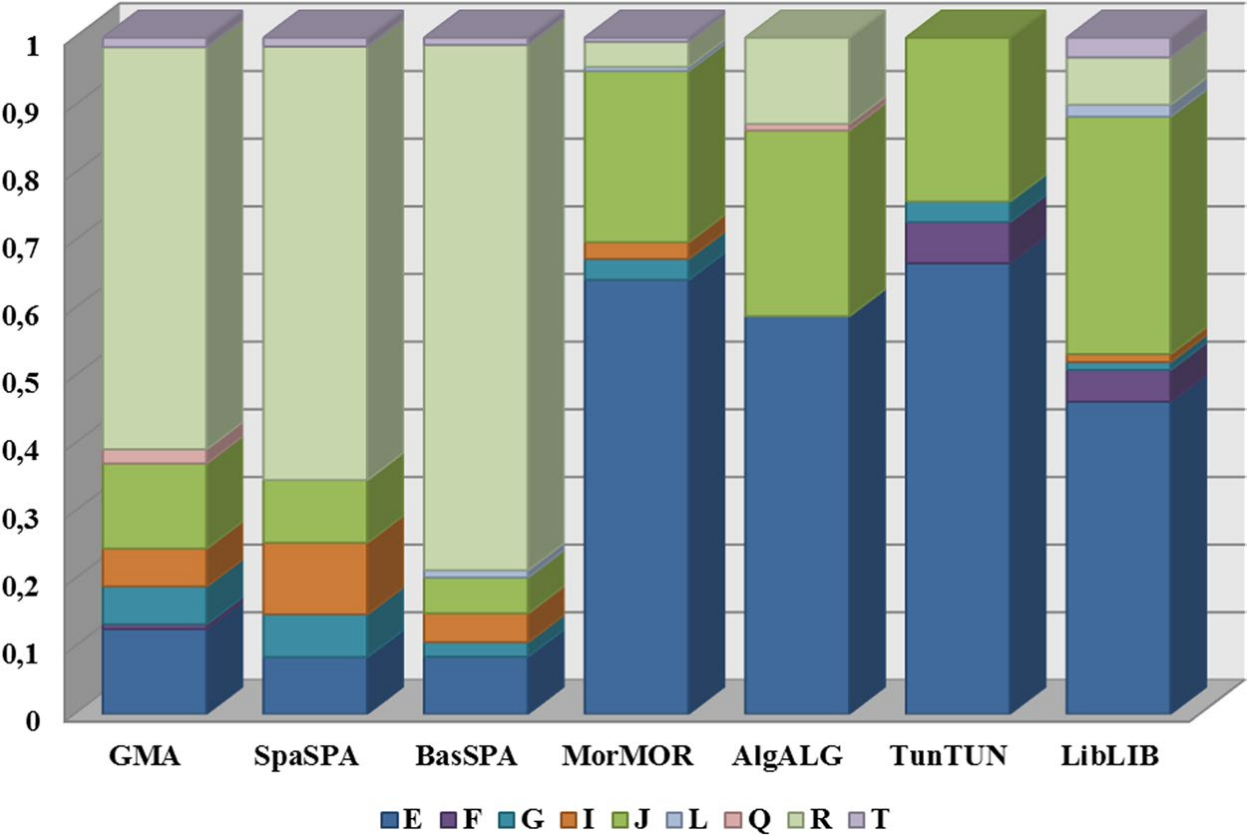
Histograms of Y chromosome haplogroup frequencies in the GMA, Spanish, and North African populations (BasSPA, Basque Country from Spain; SpaSPA, Spain; MorMOR, Morocco; AlgALG, Oran Area from Algeria; TunTUN, Tunisia; LibLIB, Libya; TurTUR, Turkey).

Based on the Y chromosome haplogroups, there was a large difference between the North African and Spanish populations—specifically the GMA population (Fig. 4). The main haplogroup in North African populations was E, which was also found in the Spanish population but at lower frequencies and similar to those in other European populations^36^. Recent studies based on the analysis of haplogroup E1b1b-M81 suggest that Andalusian Y chromosomes may derive from a single common ancestor imported from North Africa that originated in the Andalusian present-day haplotypes^12^. Analyzing the second most common Y chromosome haplogroup among North African populations (haplogroup J), the GMA population had high values of this haplogroup. However, a detailed evaluation of J subhaplogroups showed that the prevalent haplogroup in the GMA population was lineage J2a4, found along the northern coast of the Mediterranean and the Caucasus, whereas the predominant subhaplogroup in the North African populations were J1 lineages, existing at low frequencies in subpopulations of southern Europe.

The analyses of allele frequencies and genetic distances confirm the results of the haplogroup analyses. Mediterranean populations were genetically closest to the GMA population, especially the Spanish populations. The genetic data revealed that no significant African component remained in the genetic legacy of the population of the southern Iberian Peninsula compared to other Iberian and European populations, despite North African people living in the region for almost 800 years. Similar results have been recently reported. An analysis of Y-chromosome lineages in the Andalusian population demonstrated that Andalusia and other Iberian populations are related to North African populations on a larger scale than other European regions but that if South European populations, which historically have been influenced by North African populations, are included in the analysis, this influence is not significant enough^12^.

Surnames are used to enhance the genetic signals of a population structure^39,40^. In most western societies, they are transmitted through the male line and inherited as alleles^41,42^, which is their transmission should closely match the inheritance of the Y chromosome. The spectrum of surnames in this study is supported by history. Most of the surnames originated in the north and center of the Peninsula but are more frequently observed in the South.

The analysis of Y chromosomes in the GMA population indicated that the male influence of the North African inhabitants that could have remained in the population in the southern peninsula did not influence the genetic legacy of the population in this region more intensely than in other Iberian populations. After the Reconquest of the region by the Catholic Monarchs, the region was repopulated with entire families from the rest of the peninsula^3^. Although many of the Moriscos who inhabited the region were converted to Christianity and although mixed marriages might have formed, the Y chromosome lineages indicate that this phenomenon occurred at such a low frequency that its small influence prevented it from surviving the 600 years that have passed since the dissolution of the Kingdom of Granada.

## Methods

### Population samples

A total of 146 buccal cell swab samples from unrelated individuals were collected. Subjects were selected according to their self-declared affiliation to the provinces of Granada, Málaga, and Almería and their residence in the region for at least 3 generations (Supplementary Fig. 1). This study was approved by the Ethics Committee of the University of Granada (Approval Number: 885). All subjects gave their informed consent per the Declaration of Helsinki. All methods were performed in accordance with the relevant guidelines and regulations of the University of Granada.

### Surnames study

The first surnames of all 146 unrelated individuals were queried and annotated to establish the relationship between the distribution of haplotypes and haplogroups and the observed surnames. Surnames were compared with an available list of Spanish surnames of Arab origin^43^.

### DNA extraction

Genomic DNA was extracted using phenol/chloroform/isoamyl alcohol and proteinase K. The DNA was purified on Amicon^®^ 100 units (Millipore) and quantified on an 0.8% agarose gel.

### Y-STR genotyping

One hundred forty-six samples were amplified using the AmpF*l*STR^®^ Yfiler^®^ PCR Amplification kit (Applied Biosystems, Foster City, CA), per the manufacturer. Alleles were separated and detected on an Applied Biosystems ABI 310 genetic analyzer. Fragment sizes were analyzed using GeneMapper ID-X v1.1 (Applied Biosystems, Foster City, CA). The alleles were named according to the number of repeated units, based on the sequenced allelic ladder (ISFG recommendations).

All individuals with complete genotypes were deposited into the YHRD database^44^ (accession numbers YA004154–YA004156).

### Statistical analyses

Y STR allele frequencies, polymorphism information content (PIC), power of discrimination (PD), and matching probability (MP) were calculated for each locus using PowerStats v12^45^. Y chromosome haplotypes were calculated with Arlequin v3.5.1.3. Analysis of molecular variance (AMOVA) was performed with Arlequin v3.5.1.3 to determine any possible population substructure.

Y chromosome haplogroups were determined with the 23-Haplogroup Beta program (online software)^46,47^ and YPredictor by Vadim Urasin v15.0.

Network analyses were performed for individuals representing all haplogroups reducing the sample size to seventy nine individuals and individuals R1b1b2 (n = 85) to determine the most common ancestor.

Correspondence analysis with Y-STR allele frequencies from published populations (Supplementary Table 1) was performed with Statistica v9.1. Y-STR haplotype data were used to calculate pairwise genetic distances (Rst) and the corresponding p-values. The genetic distances were then used to construct a contour map with Surfer13.

## Supplementary information

Supplementary Material
