# Supplementary material for: Genetic structure in the paternal lineages of South East Spain revealed by the analysis of 17 Y-STRs
Source: Sci Rep. 2019 Mar 26;9:5234. doi: 10.1038/s41598-019-41580-9 (PMC6435739; doi:10.1038/s41598-019-41580-9)
Supplement: Supplementary file 1 — Supplementary Material [file 41598_2019_41580_MOESM1_ESM.pdf]

Genetic structure in the paternal lineages of South East Spain revealed by the analysis of 17 Y-STRs

María Saiz[1], María Jesús Álvarez-Cubero[2], José Antonio Lorente[1,3*], Juan Carlos Álvarez[1], Luis Javier Martinez-Gonzalez[3].

1. University of Granada. Laboratory of Genetic Identification. Department of Legal Medicine, Toxicology and Physical Anthropology. Faculty of Medicine. PTS. Avd. Investigación 11, 18016. Granada. Spain.
2. University of Granada. Department of Biochemistry and Molecular Biology III and Inmunology. Faculty of Medicine. PTS. Avd. Investigación 11, 18016. Granada. Spain.
3. GENYO. Centre for Genomics and Oncological Research: Pfizer / University of Granada / Andalusian Regional Government. PTS. Avenida de la Ilustración, 114. 18016 Granada, Spain

Corresponding Author: José Antonio Lorente.

Address: University of Granada. Laboratory of Genetic Identification. Department of Legal Medicine, Toxicology and Physical Anthropology. Faculty of Medicine. PTS. Avd. Investigación 11, 18016. Granada. Spain.

Telefone number: 0034958243546

Fax number: 0034 958 637 071

Email: jose.lorente@genyo.es

Supplementary Figure 1: Network for haplogroup R1b1b2 (n=85). Colour represents the different provinces; red, Granada; green, Málaga; blue, Almería. Torso is represented with darker lines.

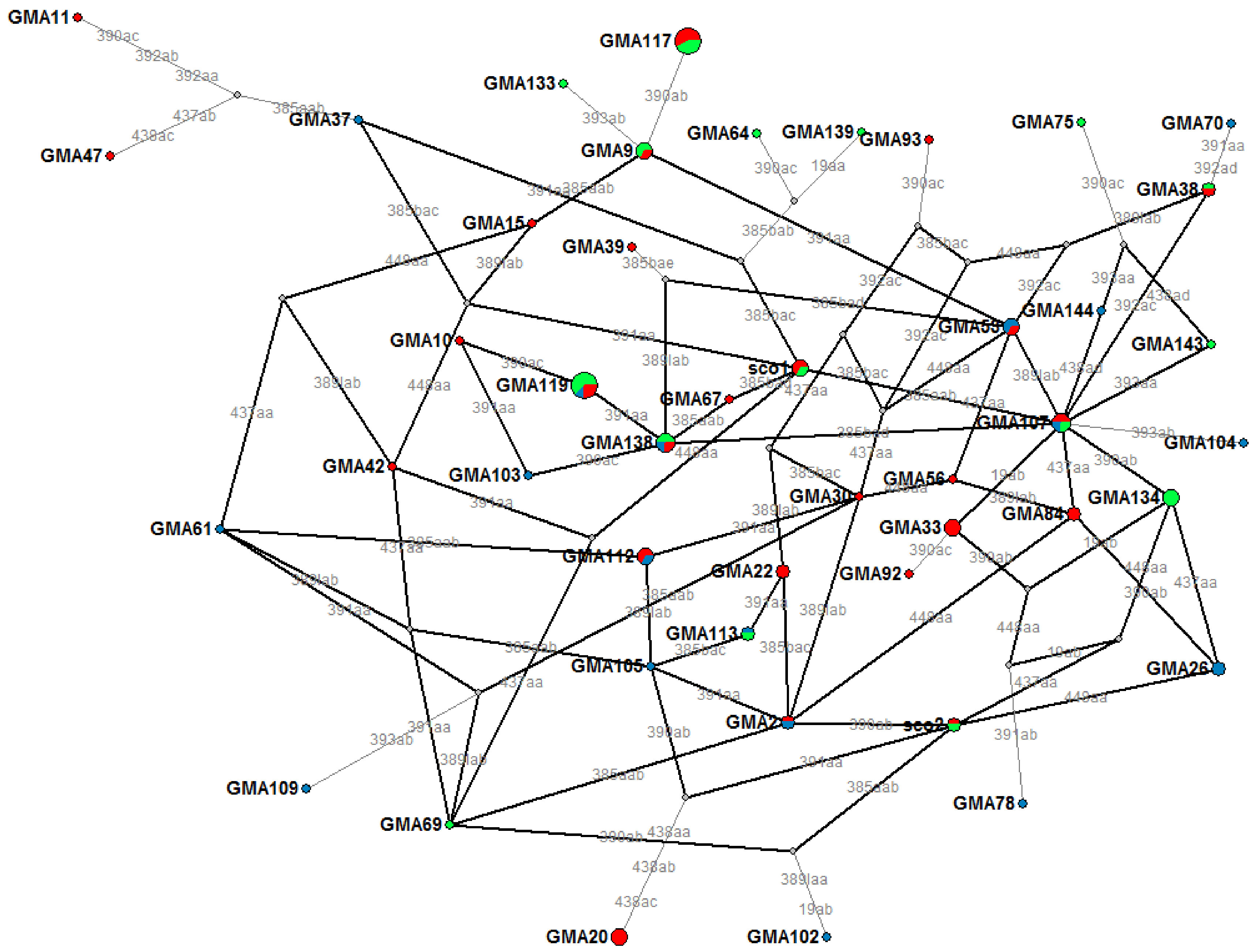

Supplementary Figure 2: Locations where the 146 samples collected. Colour represents the different provinces; red, Granada; green, Málaga; blue, Almería. Intensity of the colour represents the proportion of individuals from each location, darker colour, higher number of individuals; lighter colour, less number of individuals. Figure generated with Google My Maps.

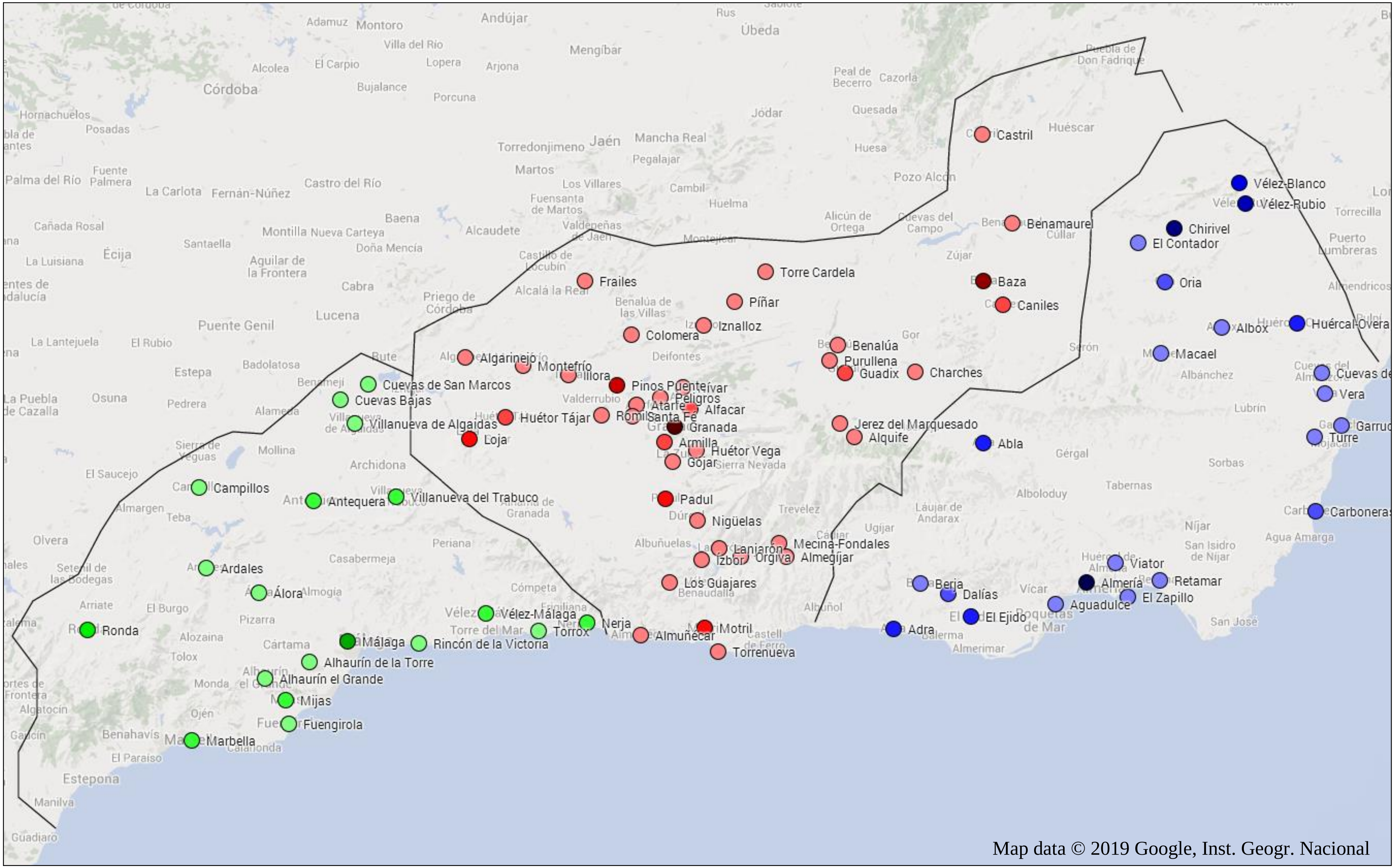

Supplementary Table 1: Complete list of populations used in the present study for comparative analysis

Population	Code	Country	Y-STRs	N	References
**Granada_Málaga_Almería**	GMA	Spain	17	146	Present study Granada YA004154, Málaga YA004155, Almería YA004156
**ALLELIC FREQUENCIES ANALYSES**					
**Morocco**	MorMOR	Morocco	17	257	Aboukhalid et al. 2010 [1]
**Figuig Oasis**	FigMOR	Morocco	17	96	Palet et al. 2010 [2]
**Oran Area**	ArgARG	Algeria	17	102	Robino et al. 2008 [3]
**Berbers from Sejenane**	BSejTUN	Tunisia	17	47	Frigi et al. 2006 [4]
**Tunisia**	TunTUN	Tunisia	15	134	Arredi et al. 2004 [5]
**Egypt**	EgyEGY	Egypt	15	76	Arredi et al. 2004 [5]
**Trípoli Region**	TriLIB	Libya	17	176	Triki-Fendri et al. 2013 [6]
**Central Anatolia**	CAnTUR	Turkey	17	140	Alakoc et al. 2010 [7]
**Basque Country**	BasSPA	Spain	23	105	Nuñez et al. 2015 [8]
**Cantabria**	CanSPA	Spain	23	98	Nuñez et al. 2015 [8]
**Barcelona**	BarSPA	Spain	17	150	Sánchez et al. 2007 [9]
**Spain**	SpaSPA	Spain	17	295	Martínez-González. Unpublished data
**North of Portugal**	NorPOR	Portugal	17	175	Pontes et al. 2007[10]
**Italy**	ItaITA	Italy	15	154	Turrina et al. 2006 [11]
**Greece**	GreGRE	Greece	11	200	Katsaloulis et al. 2013 [12]
**Croatia**	CroCRO	Croatia	17	1100	Mršić et al. 2012 [13]
**Vojvodina**	VojSER	Serbia	17	185	Veselinovic et al. 2008 [14]
**Holland**	HolHOL	Holland	36	2085	Westen et al. 2015 [15]
**Equatorial Guinea**	EqGEQG	Equatorial Guinea	16	100	Arroyo-Pardo et al. 2005 [16]
**Ovambo**	OvaNAM	Namibia	28	52	Fujihara et al. 2009 [17]
**GENETIC DISTANCES ANALYSES**					
**Algeria**	AlgALG	Algeria	17	102	YA003114 Robino et al. 2008 [3]
**Libya**	LibLIB	Libia	17	360	YA003680 Elmrghni et al. 2012 [18]; YA003083, YA003752 Triki-Fendri et al. 2013 [6]; YA003669 Ottoni et al. 2011 [19]; YA003690; YA003691
**Morocco**	MorMOR	Morocco	17	445	YA003437, YA003438, YA003439 Aboukhalid et al 2010 [1]; YA003442, YA003443 Palet et al. 2010 [2]; YA003520 Laouina et al. 2011 [20]
**Tunisia**	TunTUN	Tunisia	17	153	YA003024, YA003025, YA003026, YA003027 Cherni et al. 2005 [21]; YA003482 Ayadi et al. 2006 [22]; YA003743
**Egypt**	EgyEGY	Egypt	17	159	YA003080 Manni et al. 2002 [23]; YA003400, YA003401, YA003403, YA003404
**Turkey**	TurTUR	Turkey	17	296	YA002964 Henke et al. 2001 [24], YA003719, YA003907
**Andalusia**	AndSPA	Spain	17	149	YA004020, YA004042

**Granada**	GraSPA	Spain	17	180	YA003685 Ambrosio et al. 2012 [25]
**Huelva**	HueSPA	Spain	17	167	YA003684 Ambrosio et al. 2012 [25]
**Basque Country**	BasSPA	Spain	27	300	YA003184 García et al. 2015 [26]; YA004063
**Galicia**	GalSPA	Spain	17	89	YA003791
**Catalonia**	CatSPA	Spain	17	1388	YA004023
**Italy**	ItaITA	Italy	17	3118	YA003991 Robino et al. 2015 [27]; YA003068 Grignani et al. 2000 [28]; YA003067, YA003088, YA003090, YA003093, YA003095 Presciuttini et al. 2001 [29]; YA003891 Robino et al. 2006 [30]; YA003447 Ferri et al. 2008 [31]; YA003295 Cerri et al. 2005 [32]; YA003327 Turrina et al. 2006 [11]; YA003405 Ghiani et al. 2002 [33]; YA003262 Ferri et al. 2009 [34]; YA003069 Onofri et al. 2007 [35]; YA003471 Verzeletti et al. 2009 [36]; YA003490, YA003491 Rodríguez et al. 2009 [37]; YA003721, YA003722, YA003723, YA003724, YA003725 YA003726 YA003744 Brisighelli et al. 2012 [38]; YA003730 Piglionica et al. 2013 [39]; YA003974; YA003786; YA003873; YA003435; YA004045; YA003926; YA003982; YA003983;
**Portugal**	PorPOR	Portugal	17	534	YA003125 Fernandes et al. 2001 [40]; YA003016 González Neira et al. 2000 [41]; YA003145 & YA003146 Carvalho et al. 2003 [42]; YA003015 & YA003017 Alves et al. 2007 [43]; YA003293
**Greece**	GreGRE	Greece	17	559	YA003127 Parreira et al. 2002 [44]; YA003097, YA003098, YA003099, YA003100, YA003101, YA003102, YA003103, Robino et al. 2004 [45]; YA003325 Bosch et al. 2006 [46]; YA003465 Kovatsi et al. 2009 [47]; YA004096, YA003647 Katsaloulis et al. 2013 [12], YA003862; YA004097
**Nigeria**	NigNIG	Nigeria	17	135	YA003874; YA003940; YA003973.
**Somalia**	SomSOM	Somalia	17	172	YA003284 Hallenberg et al. 2005 [48]

Supplementary references

1. Aboukhalid, R. *et al.* Haplotype frequencies for 17 Y-STR loci (AmpFlSTR®Y-filer™) in a Moroccan population sample. *Forensic Sci. Int. Genet.* **4,** e73–e74 (2010).
2. Palet, L. *et al.* Y-STR genetic diversity in Moroccans from the Figuig oasis. *Forensic Sci. Int. Genet.* **4,** (2010).
3. Robino, C. *et al.* Analysis of Y-chromosomal SNP haplogroups and STR haplotypes in an Algerian population sample. *Int. J. Legal Med.* **122,** 251–255 (2008).
4. Frigi, S. *et al.* Data for Y-chromosome haplotypes defined by 17 STRs (AmpFLSTR® Yfiler™) in two Tunisian Berber communities. *Forensic Sci. Int.* **160,** 80–83 (2006).
5. Arredi, B. *et al.* A Predominantly Neolithic Origin for Y-Chromosomal DNA Variation in North Africa. *Am. J. Hum. Genet.* **75,** 338–345 (2004).
6. Triki-Fendri, S. *et al.* Population genetics of 17 Y-STR markers in West Libya (Tripoli region). *Forensic Sci. Int. Genet.* **7,** e59-61 (2013).
7. Alakoc, Y. D. *et al.* Y-chromosome and autosomal STR diversity in four proximate settlements in Central Anatolia. *Forensic Sci. Int. Genet.* **4,** (2010).
8. Nuñez, C. *et al.* Highly discriminatory capacity of the PowerPlex(®) Y23 System for the study of isolated populations. *Forensic Sci. Int. Genet.* **17,** 104–7 (2015).
9. Sánchez, C. *et al.* Haplotype frequencies of 16 Y-chromosome STR loci in the Barcelona metropolitan area population using Y-Filer™ kit. *Forensic Sci. Int.* **172,** 211–217 (2007).
10. Pontes, M. L., Cainé, L., Abrantes, D., Lima, G. & Pinheiro, M. F. Allele frequencies and population data for 17 Y-STR loci (AmpFℓSTR® Y-filer™) in a Northern Portuguese population sample. *Forensic Sci. Int.* **170,** 62–67 (2007).
11. Turrina, S., Atzei, R. & De Leo, D. Y-chromosomal STR haplotypes in a Northeast Italian population sample using 17plex loci PCR assay. *Int. J. Legal Med.* **120,** 56–59 (2006).
12. Katsaloulis, P., Tsekoura, K., Vouropoulou, M. & Miniati, P. Genetic population study of 11 Y chromosome STR loci in Greece. *Forensic Sci. Int. Genet.* **7,** e56-8 (2013).
13. Mršić, G. *et al.* Croatian national reference Y-STR haplotype database. *Mol. Biol. Rep.* **39,** 7727–41 (2012).
14. Veselinovic, I. S. *et al.* Allele frequencies and population data for 17 Y-chromosome STR loci in a Serbian population sample from Vojvodina province. *Forensic Sci. Int.* **176,** e23–e28 (2008).
15. Westen, A. a. *et al.* Analysis of 36 Y-STR marker units including a concordance study among 2085 Dutch males. *Forensic Sci. Int. Genet.* **14,** 174–181 (2015).
16. Arroyo-Pardo, E. *et al.* Genetic variability of 16 Y-chromosome STRs in a sample from Equatorial Guinea (Central Africa). *Forensic Sci. Int.* **149,** 109–113 (2005).
17. Fujihara, J. *et al.* Allele frequencies and haplotypes for 28 Y-STRs in Ovambo population. *Leg. Med.* **11,** 205–208 (2009).
18. Elmrghni, S., Coulson-Thomas, Y. M., Kaddura, M., Dixon, R. A. & Williams, D. R. Population genetic data for 17 Y STR markers from Benghazi (East Libya). *Forensic Sci. Int. Genet.* **6,** 224–7 (2012).
19. Ottoni, C. *et al.* Deep into the roots of the Libyan Tuareg: A genetic survey of

their paternal heritage. *Am. J. Phys. Anthropol.* **145,** 118–124 (2011).
20. Laouina, A. *et al.* Allele frequencies and population data for 17 Y-STR loci (The AmpFlSTR® Y-filer™) in Casablanca resident population. *Forensic Sci. Int. Genet.* **5,** e1-3 (2011).
21. Cherni, L. *et al.* Y-chromosomal STR haplotypes in three ethnic groups and one cosmopolitan population from Tunisia. *Forensic Sci. Int.* **152,** 95–9 (2005).
22. Ayadi, I., Ammar-Keskes, L. & Rebai, A. Haplotypes for 13 Y-chromosomal STR loci in South Tunisian population (Sfax region). *Forensic Sci. Int.* **164,** 249–53 (2006).
23. Manni, F. *et al.* Y-chromosome analysis in Egypt suggests a genetic regional continuity in Northeastern Africa. *Hum. Biol.* **74,** 645–58 (2002).
24. Henke, J. *et al.* Application of Y-chromosomal STR haplotypes to forensic genetics. *Croat. Med. J.* **42,** 292–7 (2001).
25. Ambrosio, B. *et al.* Y-STR genetic diversity in autochthonous Andalusians from Huelva and Granada provinces (Spain). *Forensic Sci. Int. Genet.* **6,** e66–e71 (2012).
26. García, O. *et al.* Data for 27 Y-chromosome STR loci in the Basque Country autochthonous population. *Forensic Sci. Int. Genet.* (2015). doi:10.1016/j.fsigen.2015.09.010
27. Robino, C. *et al.* Development of an Italian RM Y-STR haplotype database: Results of the 2013 GEFI collaborative exercise. *Forensic Sci. Int. Genet.* **15,** 56–63 (2015).
28. Grignani, P., Peloso, G., Fattorini, P. & Previderè, C. Highly informative Y-chromosomal haplotypes by the addition of three new STRs DYS437, DYS438 and DYS439. *Int. J. Legal Med.* **114,** 125–9 (2000).
29. Presciuttini, S. *et al.* Y-chromosome haplotypes in Italy: the GEFI collaborative database. *Forensic Sci. Int.* **122,** 184–8 (2001).
30. Robino, C. *et al.* Y-chromosomal STR haplotypes in Sicily. *Forensic Sci. Int.* **159,** 235–40 (2006).
31. Ferri, G. *et al.* Male haplotypes and haplogroups differences between urban (Rimini) and rural area (Valmarecchia) in Romagna region (North Italy). *Forensic Sci. Int.* **175,** 250–5 (2008).
32. Cerri, N., Verzeletti, A., Bandera, B. & De Ferrari, F. Population data for 12 Y-chromosome STRs in a sample from Brescia (northern Italy). *Forensic Sci. Int.* **152,** 83–7 (2005).
33. Ghiani, M. E. & Vona, G. Y-chromosome-specific microsatellite variation in a population sample from Sardinia (Italy). *Coll. Antropol.* **26,** 387–401 (2002).
34. Ferri, G., Alù, M., Corradini, B., Radheshi, E. & Beduschi, G. Slow and fast evolving markers typing in Modena males (North Italy). *Forensic Sci. Int. Genet.* **3,** e31-3 (2009).
35. Onofri, V. *et al.* Y-chromosome genetic structure in sub-Apennine populations of Central Italy by SNP and STR analysis. *Int. J. Legal Med.* **121,** 234–7 (2007).
36. Verzeletti, A. *et al.* Population data for 15 autosomal STRs loci and 12 Y chromosome STRs loci in a population sample from the Sardinia island (Italy). *Leg. Med. (Tokyo).* **11,** 37–40 (2009).
37. Rodríguez, V. *et al.* Genetic sub-structure in western Mediterranean populations revealed by 12 Y-chromosome STR loci. *Int. J. Legal Med.* **123,** 137–41 (2009).
38. Brisighelli, F. *et al.* Patterns of Y-STR variation in Italy. *Forensic Sci. Int. Genet.* **6,** 834–839 (2012).
39. Piglionica, M. *et al.* Population data for 17 Y-chromosome STRs in a sample

from Apulia (Southern Italy). *Forensic Sci. Int. Genet.* **7,** e3-4 (2013).
40. Fernandes, A. T., Brehm, A., Gusmão, L. & Amorim, A. Y-chromosome STR haplotypes in the Madeira archipelago population. *Forensic Sci. Int.* **122,** 178–80 (2001).
41. González-Neira, A. *et al.* Distribution of Y-chromosome STR defined haplotypes in Iberia. *Forensic Sci. Int.* **110,** 117–26 (2000).
42. Carvalho, M. *et al.* Y-chromosome STR haplotypes in two population samples: Azores Islands and Central Portugal. *Forensic Sci. Int.* **134,** 29–35 (2003).
43. Alves, C., Gomes, V., Prata, M. J., Amorim, A. & Gusmão, L. Population data for Y-chromosome haplotypes defined by 17 STRs (AmpFlSTR YFiler) in Portugal. *Forensic Sci. Int.* **171,** 250–255 (2007).
44. Parreira, K. S., Lareu, M. V., Sánchez-Diz, P., Skitsa, I. & Carracedo, A. DNA typing of short tandem repeat loci on Y-chromosome of Greek population. *Forensic Sci. Int.* **126,** 261–4 (2002).
45. Robino, C. *et al.* Y-chromosomal STR haplotypes in a population sample from continental Greece, and the islands of Crete and Chios. *Forensic Sci. Int.* **145,** 61–4 (2004).
46. Bosch, E. *et al.* Paternal and maternal lineages in the Balkans show a homogeneous landscape over linguistic barriers, except for the isolated Aromuns. *Ann. Hum. Genet.* **70,** 459–87 (2006).
47. Kovatsi, L., Saunier, J. L. & Irwin, J. A. Population genetics of Y-chromosome STRs in a population of Northern Greeks. *Forensic Sci. Int. Genet.* **4,** e21-2 (2009).
48. Hallenberg, C., Nielsen, K., Simonsen, B., Sanchez, J. & Morling, N. Y-chromosome STR haplotypes in Danes. *Forensic Sci. Int.* **155,** 205–10 (2005).
